# Papillary Renal Cell Carcinoma With Widespread Metastases in an Arabian Mare

**DOI:** 10.1002/vms3.70281

**Published:** 2025-04-02

**Authors:** Omid Azari, Seyed Mahdi Ghamsari, Ali Roustaei, Diba Golchin, Farid Baharloo, Mohammad Javaheri, Negar Valizadeh

**Affiliations:** ^1^ Department of Surgery and Radiology Faculty of Veterinary Medicine University of Tehran Tehran Iran; ^2^ Department of Pathology Faculty of Veterinary Medicine University of Tehran Tehran Iran; ^3^ Large Animal Internal Medicine Specialist Equine Practitioner Isfahan Iran

**Keywords:** colic, horse, kidney, renal cell carcinoma

## Abstract

This report describes a case of massive renal cell carcinoma (RCC) causing mild chronic colic in a 20‐day‐postpartum Arabian mare. The mare presented with deteriorating abdominal pain following normal parturition. Clinical examination revealed tachycardia, tachypnoea, weak intestinal motility and pale mucosal membranes. Rectal examination identified a very large mass in the caudal part of the abdominal cavity, and abdominal ultrasound examination revealed a huge space‐occupying mass with renal architecture. During midline exploratory laparotomy, a massive tumour was observed in the anatomical location of the left kidney, with numerous adhesions to abdominal organs. Due to the severity of the condition and intra‐abdominal spread of the neoplasm, the horse was euthanized intraoperatively. Upon necropsy, a 23‐kg mass was found in the left kidney, along with widespread metastases to the liver, diaphragm and lungs. Histopathological examination confirmed primary and metastatic papillary RCC. This case highlights that colic should be considered a potential symptom of neoplastic lesions within the abdomen.

## Graphical abstract

An 8‐year‐old Arabian mare presented with mild abdominal pain and weight loss. A huge papillary renal cell carcinoma with widespread metastases was identified via exploratory laparotomy, followed by histopathologic examination. Colic must be considered as one of the symptoms of intra‐abdominal neoplastic lesions.

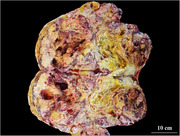

## 1 Introduction

Renal carcinoma, also known as renal cell carcinoma (RCC) or renal adenocarcinoma, is the most frequent renal neoplasm in humans and the most commonly diagnosed primary neoplasm of the upper urinary tract in dogs and horses (Wise et al. 2009). Although primary renal neoplasia is uncommon in horses, when confirmed, it is often malignant, characterized by highly invasive and metastatic lesions (Oosterlinck et al. 2011).

Sex and breed are not considered predisposing factors for RCC, and horses of all ages can develop this condition. Although both kidneys can be affected, the tumour often occurs unilaterally. Clinical diagnosis is challenging due to the variability in clinical signs, which may not always involve the urinary tract (Ramirez and Seahorn 1996). Clinical signs can include cow dung‐like faecal consistency, increased borborygmi on abdominal auscultation, abdominal distension, limb edema, poor body condition, normal capillary refill time, normal respiratory and heart rates, pink mucous membranes, weight loss, haematuria, pyrexia, anaemia, colic and the presence of a mass on rectal examination (Schott et al. 2019; Birkmann et al. 2016).

Given the limitations of radiography in examining abdominal organs in large animal practice, ultrasonography is the preferred diagnostic tool for detecting intra‐abdominal lesions (Ramirez and Seahorn 1996). The overall success of surgical treatment for RCC is limited due to its aggressive nature, vague symptoms that often lead to delayed diagnosis, and the tendency for widespread metastasis (Wise et al. 2009; Hilton et al. 2008). Moreover, in such cases, the recurrence rate following nephrectomy has been reported to range from 20% to 40% (Chin et al. 2006).

This article describes the clinical signs, diagnostic workup, necropsy findings and histopathological features of a case of massive papillary RCC with widespread intra‐abdominal metastases in a 20‐day postpartum Arabian mare.

### 1.1 Anamnesis and Clinical Examination

An 8‐year‐old Arabian mare with a history of normal parturition 20 days earlier, mild abdominal pain, weight loss over the past 2 months and postpartum exacerbation of symptoms was referred to the Veterinary Teaching Hospital of the University of Tehran. The mare's condition had progressively worsened, prompting further diagnostic investigation and management.

### 1.2 Clinical Findings

The mare was depressed but alert, exhibiting decreased appetite and loose faeces. Physical examination revealed caecal and colonic hypomotility. Cardiac and respiratory rates were 55 beats/min and 35 breaths/min, respectively, indicating moderate tachycardia and tachypnoea. The mare's rectal temperature was 37.8°C, and her mucus membranes appeared mildly pale, with a capillary refill time of 3 seconds. Rectal examination revealed a large, firm space‐occupying mass in the caudal part of the abdominal cavity, with evidence of extension into the pelvic cavity. Midline abdominocentesis yielded sanguineous peritoneal fluid, and cytological analysis confirmed the presence of numerous red blood cells and haemolysis. These findings raised concerns about intraabdominal haemorrhage or a neoplastic process.

### 1.3 Laboratory Findings

Haematological, biochemical and urinary parameters were within normal limits as follows (Weiss and Wardrop 2011; Duncan and Prasse 1981): calcium, 11.7 mg/dL; urea, 29 mg/dL; blood urea nitrogen, 13.5 mg/dL; creatinine, 0.93 mg/dL; haematocrit, 53%; haemoglobin, 18.2 g/dL; red blood cells, 11 × 10^6^/µL; mean corpuscular volume, 52 fL; mean corpuscular haemoglobin, 16.1 pg; mean corpuscular haemoglobin concentration, 31.5 g/dL; platelets, 367 × 10^3^/µL; white blood cells, 5800 × 10^3^/µL; neutrophils, 64%; lymphocytes, 13%; band cells, 17%; monocytes, 2%; eosinophils, 3%; basophils, 1%; plasma protein, 7.6 g/dL; total protein, 7.4 g/dL; and fibrinogen, 0.4 g/dL.

### 1.4 Ultrasonography Findings

**FIGURE 1:**
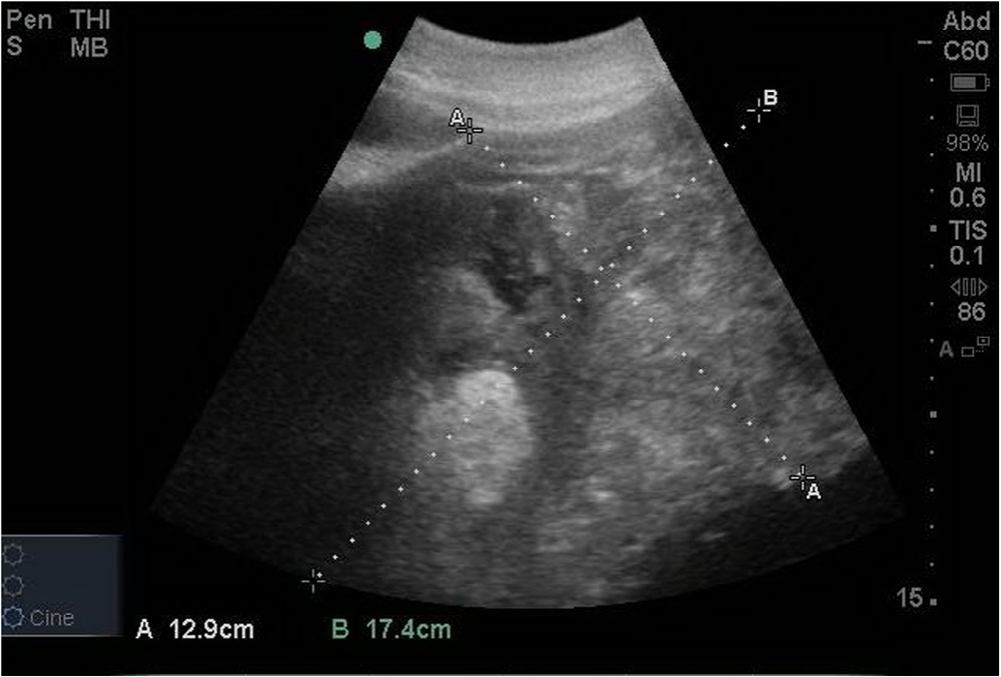
Ultrasound image depicting a large, heterogeneous, hyperechoic mass with regions of mineralization and a cavitary anechoic cystic structure at the level of the left kidney, extending caudal to the 17th and 18th left thoracic ribs. The maximum size of the kidney visualized in this section is 12.9 × 17.4 cm.

An ultrasound device (M‐Turbo, SonoSite Inc., Bothell, Washington, USA) equipped with a 1–5‐MHz convex probe was used for transcutaneous abdominal ultrasonography of both paralumbar regions. In the left nephrosplenic window, a large, well‐defined mass with mixed‐echogenicity was observed at the level of the left kidney. The mass contained mineralized regions and cavitary structures and was attached to the spleen. Additionally, hypoechoic free fluid was visible in the peritoneal cavity, along with a thickened small bowel wall measuring 1.2 cm, indicative of peritonitis (Figure 1). These findings further supported the suspicion of a neoplastic process with secondary complications such as peritoneal effusion and inflammation.

### 1.5 Surgical Treatment

Based on the findings of physical and paraclinical examinations, a massive renal tumour was suspected, prompting a recommendation for emergency exploratory celiotomy, to which the owner consented. Following this decision, general anaesthesia was induced by intravenous injection of 1.1 mg/kg xylazine (Bioveta, Czech Republic), 2.2 mg/kg ketamine (Alfasan, Holland) and 0.2 mg/kg diazepam (Caspian, Iran) and was maintained with isoflurane (Piramal, India) in oxygen. An exploratory laparotomy was then performed through a midline incision. In the abdominal cavity, a large mass was observed in the left kidney, with extensive adhesions to both the small and large intestines. Additionally, a significant volume of dark yellow peritoneal fluid was drained. Due to the large size of the renal mass, multicentric metastases, intra‐abdominal adhesions and a high risk of intraoperative haemorrhage, nephrectomy was deemed infeasible; hence, euthanasia was discussed with the owner. After written informed consent was obtained from the owner, the patient was euthanized under general anaesthesia.

### 1.6 Necropsy Findings

**FIGURE 2:**
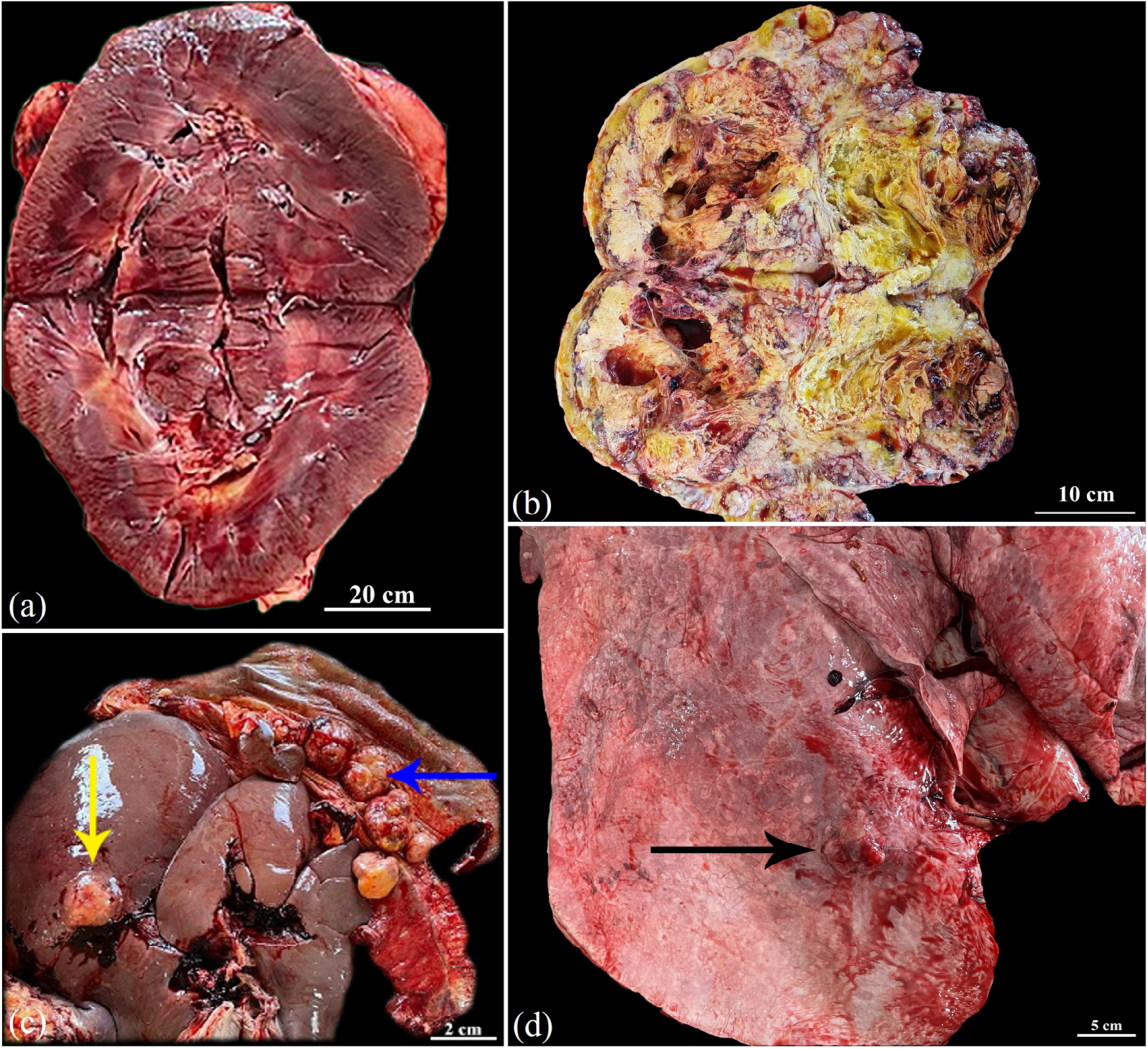
Renal cell carcinoma. (a) Right kidney appearing normal. (b) Longitudinally incised left kidney with a renal mass weighing 23 kg. (c and d) Numerous sessile and pedunculated masses of varying sizes scattered throughout the peritoneum, most prominently on the liver (yellow arrow), diaphragm (blue arrow) and lungs (black arrow).

At necropsy, the right kidney (Figure 2a) and urinary bladder appeared normal. In contrast, a firm, non‐encapsulated mass, grey to cream in colour, and weighing 23 kg, was found in the left kidney (Figure 2b). The mass had an irregular surface and was adherent to the intestines. Extensive haemorrhagic foci were observed on the spleen, and multiple metastatic nodules were identified in the liver, diaphragm and lungs (Figure 2c,d). Following macroscopic examination, tissue samples were obtained from both kidneys, metastatic lesions and other normal‐appearing organs. The specimens were fixed in 10% neutral‐buffered formalin, processed, embedded in paraffin and sectioned at 5‐µm thickness. They were then stained with hematoxylin and eosin (H&E) and PAX8 immunohistochemistry (IHC), and examined under a light microscope (Olympus CX33).

### 1.7 Histopathology Findings

**FIGURE 3:**
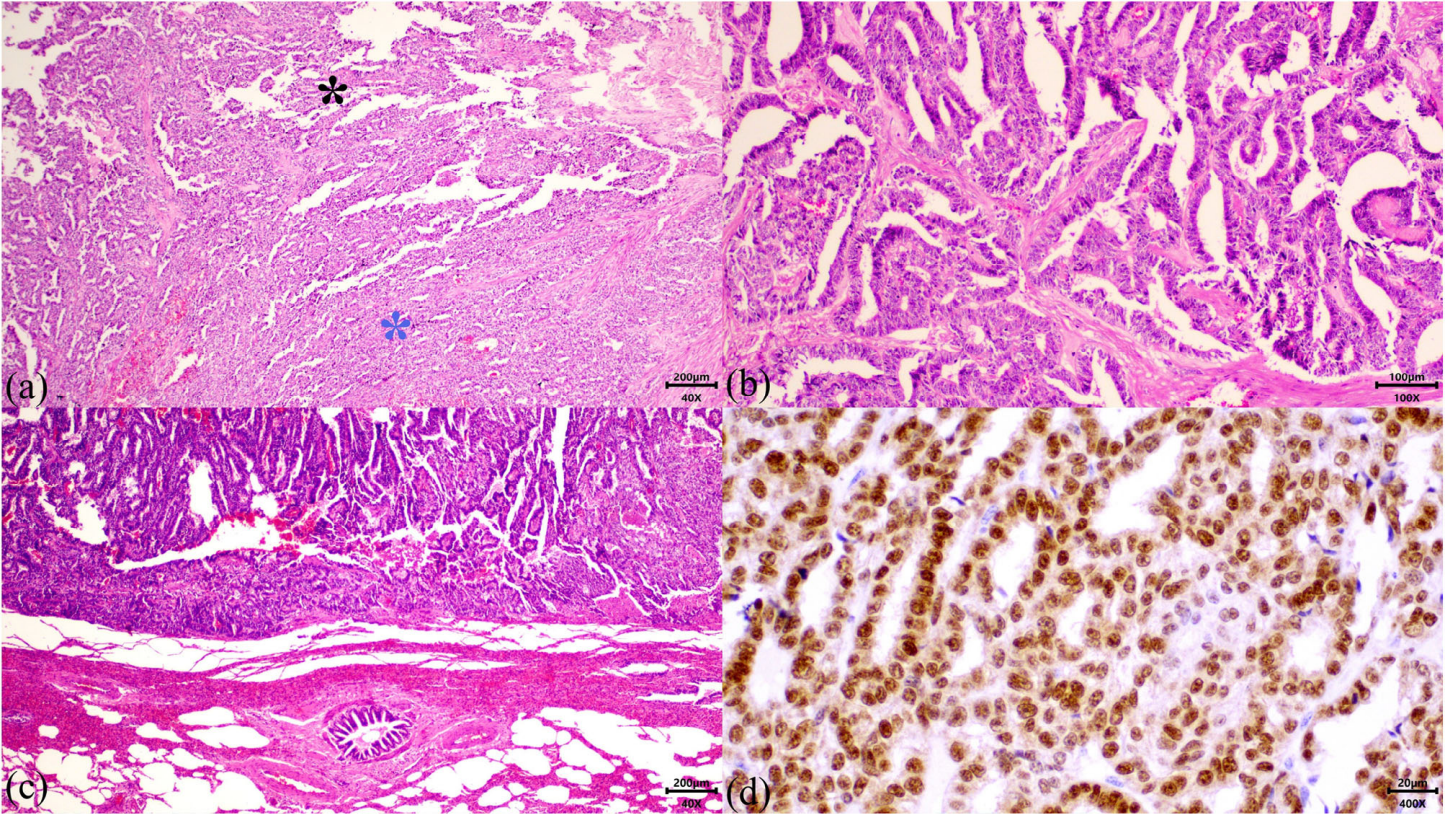
Microphotographs of the primary renal cell carcinoma, its pulmonary metastases and immunohistochemical examination. (a) Kidney. Renal cell carcinoma with both papillary (black asterisk) and tubular (blue asterisk) patterns. H&E. Bar = 200 µm. (b) Diaphragmatic metastatic mass. Papillary arrangement of the metastatic neoplastic cells mimicking those of the primary neoplasm. H&E. Bar = 100 µm. (c) Pulmonary metastatic mass. Metastatic neoplastic cells are depicted in the upper portion of the picture, while hyperaemic pulmonary parenchyma is evident in the lower aspect of the figure. H&E. Bar = 200 µm. (d) Pax‐8 strong nuclear immunoreactivity of the neoplastic cells. IHC. Bar = 20 µm.

Histopathological examination of the left kidney revealed malignant pleomorphic neoplastic epithelial cells arranged predominantly in papillae and sparsely in tubules, with focally extensive necrosis, haemorrhage and fibrin deposition. Thus, a diagnosis of primary papillary RCC was established. Similar cellular components were observed in the lungs, liver and diaphragm, indicating metastatic foci. Severe haemorrhage was the only lesion noted in the spleen. Histopathological examination of other organs, including the right kidney, revealed no neoplastic cells (Figure 3a–c). PAX8 (Rabbit Monoclonal Antibody, ab227707, Abcam, Cambridge, UK) immunostaining was performed at a dilution of 1:100. Strong diffuse nuclear immunoreactivity (Figure 3d) of the neoplastic cells confirmed the initial diagnosis.

## 2 Discussion

Renal tumours are uncommon in horses but have a notably high potential for malignancy. They account for approximately 0.11% of all tumours in horses, with RCC cases comprising half of these neoplasms (Haschek et al. 1981). In a study of 1,069 necropsied horses, renal tumours were identified in 20 carcasses (1.87%), 15 of which were primary cases (Vienenkötter et al. 2017). In cats and dogs, the occurrence of secondary renal neoplasms is observed to be two to seven times more frequent than that of primary renal neoplasms. Lymphosarcoma is typically recognized as the most frequently observed secondary renal tumour across various species, a finding that was also corroborated with the study of Vienenkötter et al. (2017), Meuten (2002), and Mooney et al. (1987).

All horses exhibiting renal tumours were over the age of 10 years with the exception of a nephroblastoma reported in a foal (Vienenkötter et al. 2017). This age distribution contrasts with findings from another study involving 15 horses with primary renal tumours, in which nearly half were under 10 years of age, which is consistent with our study (Traub‐Dargatz 1998). According to this study, there was no clear breed preference observed. Among the 14 horses diagnosed with renal primary neoplasia, eight were female, indicating that, contrary in dogs and humans, there was no male's predominance (Baskin and De Paoli 1977; Lopez‐Beltran et al. 2006).

Given their nonspecific symptoms and late diagnosis, renal neoplasms are generally challenging to treat (Knowles et al. 2008). Various studies have reported clinical signs such as weight loss, colic, haematuria, ascites, pyrexia, polyuria and polydipsia in association with renal neoplasms (Haschek et al. 1981; Brown and Holt 1985).

In the current study, a complete blood count revealed normal results. Since RCC frequently occurs unilaterally, azotaemia is rarely observed in affected patients (Traub‐Dargatz 1998). Furthermore, serum urea and creatinine levels often remain normal in horses with renal carcinoma due to the reserve functional capacity of the contralateral kidney (Knowles et al. 2008).

While a previous study found metastases in approximately 70% of equine RCC cases (Wise et al. 2009), Vienenkötter et al. (2017) reported a significantly lower metastasis rate of 11% (Vienenkötter et al. 2017). In horses with RCC, the lungs and liver are the most common sites of metastasis (Brown and Holt 1985). Additionally, RCC metastases have been documented in the contralateral kidney, heart, pancreas, intestines, adrenal glands, myocardium and lymph nodes (Rhind et al. 1999; Romero et al. 2022). Moreover, Romero et al. (2022) reported RCC metastasis to both uveal structures in a blind horse (Romero et al. 2022). Research has also shown that intraosseous metastasis of RCC can occur in horses, with specific cases involving the maxilla, olecranon and humerus (Rumbaugh et al. 2003; Rhind et al. 1999; Young 2008). In the present study, metastatic RCC lesions were identified in the liver and lungs, which are typical sites of metastasis, while the contralateral kidney was spared. Additionally, multiple metastatic lesions were observed on the diaphragm, a rare finding in horses with RCC (Rumbaugh et al. 2003). PAX8 is a transcription factor known as a specific and sensitive marker for human RCCs and ovarian neoplasms, especially serous and primary ovarian tumours (Tacha et al. 2011; Kim et al. 2023). In veterinary medicine, PAX8 has been proven to be a reliable, specific and sensitive immunohistochemical diagnostic marker for primary and metastatic RCCs of various histological types in dogs (Peat et al. 2017; Bellini et al. 2020) and cats (Ramos‐Vara et al. 2017; Wu et al. 2022). To the authors’ knowledge, this is the first documentation of PAX8 immunostaining in equine RCC.

Abdominocentesis is a diagnostic procedure that can be used to evaluate colic and abdominal neoplasia. The presence of neoplastic cells in the peritoneal fluid is a highly specific, but insensitive indicator of abdominal tumours (Knowles et al. 2008). In our study, abdominocentesis revealed only hemoperitoneum and haemolysis. A previous study reported that intra‐abdominal haemorrhage was detected via abdominocentesis in 21% of horses with RCC (Wise et al. 2009). In our patient, the exact origin of intra‐abdominal haemorrhage could not be determined during necropsy. However, rupture of the affected kidney is the most common source of abdominal haemorrhage in horses with RCC (Mesquita et al. 2016).

The prognosis for equine RCC is generally poor (Knowles et al. 2008). Although nephrectomy is the treatment of choice for RCC, its overall success is often limited by the large size of the tumours and their adhesion to surrounding organs. As a result, surgical removal is usually impossible due to uncontrollable intraoperative haemorrhage. Widespread metastases to the lungs and liver further indicate that renal carcinomas are typically untreatable (Schott et al. 2019). Sudden death due to acute intraoperative haemorrhage has also been reported in horses with RCC (Knowles et al. 2008).

## 3 Conclusion

The present report described nonspecific clinical signs and laboratory findings associated with RCC in an 8‐year‐old Arabian mare. Similar to many other abdominal tumours, renal carcinomas often remain clinically asymptomatic until they are significantly advanced. Consistent with previous reports, the present case exhibited widespread distant metastases, including to the diaphragm‐ a rare metastatic site.

In conclusion, routine clinical examinations and comprehensive annual ultrasonography during the breeding season are recommended to facilitate early detection of neoplastic masses, thereby improving the likelihood of successful treatment. Additionally, veterinary care should be sought immediately in cases of colic, as it may be a symptom of intra‐abdominal neoplastic lesions. Additionally, PAX8 is recommended as a useful diagnostic immunomarker for diagnosis and distinguishing RCC in horses.

## Author Contributions

**Omid Azari**: conceptualization, formal analysis, investigation, supervision. **Seyed Mahdi Ghamsari**: visualization, methodology. **Ali Roustaei**: investigation, data curation, writing – original draft preparation. **Diba Golchin**: investigation, resources, visualization, writing – review and editing. **Farid Baharlo**: investigation. **Mohammad Javahheri**: investigation. **Negar Valizadeh**: writing – original draft preparation, writing – review and editing.

## Ethics Statement

The authors have nothing to report.

## Conflicts of Interest

The authors declare no conflicts of interest.

### Peer Review

The peer review history for this article is available at https://www.webofscience.com/api/gateway/wos/peer‐review/10.1002/vms3.70281

## Data Availability

Data are available from the corresponding author upon reasonable request.
